# Construction of a Comprehensive Diagnostic Scoring Model for Prostate Cancer Based on a Novel Six-Gene Panel

**DOI:** 10.3389/fgene.2022.831162

**Published:** 2022-04-26

**Authors:** Yunfeng Liu, Simei Qiu, Dongshan Sun, Ting Xiong, Qiuling Xiang, Quhuan Li

**Affiliations:** ^1^ School of Bioscience and Bioengineering, South China University of Technology, Guangzhou, China; ^2^ Guangdong Provincial Engineering and Technology Research Center of Biopharmaceuticals, South China University of Technology, Guangzhou, China; ^3^ Zhongshan School of Medicine, Sun Yat-sen University, Guangzhou, China

**Keywords:** m^6^A modification, prostate cancer, diagnostic scoring model, METTL14, tumor microenvironment

## Abstract

Accumulating evidence indicates that the N6-methyladenosine (m^6^A) modification plays a critical role in human cancers. Given the current understanding of m^6^A modification, this process is believed to be dynamically regulated by m^6^A regulators. Although the discovery of m^6^A regulators has greatly enhanced our understanding of the mechanism underlying m^6^A modification in cancers, the function and role of m^6^A in the context of prostate cancer (PCa) remain unclear. Here, we aimed to establish a comprehensive diagnostic scoring model that can act as a complement to prostate-specific antigen (PSA) screening. To achieve this, we first drew the landscape of m^6^A regulators and constructed a LASSO-Cox model using three risk genes (METTL14, HNRNP2AB1, and YTHDF2). Particularly, METTL14 expression was found to be significantly related to overall survival, tumor T stage, relapse rate, and tumor microenvironment of PCa patients, showing that it has important prognostic value. Furthermore, for the sake of improving the predictive ability, we presented a comprehensive diagnostic scoring model based on a novel 6-gene panel by combining with genes found in our previous study, and its application potential was further validated by the whole TCGA and ICGC cohorts. Our study provides additional clues and insights regarding the treatment and diagnosis of PCa patients.

## Introduction

As the second most frequent type of cancer in men, prostate cancer (PCa) is gradually becoming a major clinical burden (Siegel et al., 2016). Typically, patients with localized PCa exhibit long-term survival, and a large number of patients develop bone metastasis (Liao et al., 2006). However, when this disease progresses from metastatic to castrate-resistant PCa (CRPC), the mortality rate of PCa patients is increased significantly in the subsequent 2–3 years (Kohaar et al., 2019). It is estimated that over 350,000 people die from PCa each year and that the number of newly diagnosed cases for PCa is increasing worldwide (Bray et al., 2018).

Over the past decade, research on PCa has developed rapidly, particularly with respect to the development of new treatment methods and understanding of the underlying mechanisms. Despite this, a number of important clinical issues remain unresolved, including the identification of reliable biomarkers as supplement to prostate-specific antigen (PSA) screening for PCa (Attard et al., 2016). The primary disadvantage of screening for PSA, which is widely used as a biomarker for PCa, is its low specificity and poor diagnostic value (Barry, 2009). It is therefore vital and urgent to discover new biomarkers that can be used for the clinical diagnosis and prognosis of PCa.

As the most common type of RNA methylation modification, N6-methyladenosine (m^6^A) has recently become a research hotspot in the life sciences and has received extensive attention worldwide, particularly in the context of cancer (Deng et al., 2018). It is well established that m^6^A is a dynamic and reversible RNA modification, and its modification level is dynamically regulated by different types of m^6^A regulators, including demethylases (“eraser”), methyltransferases (“writer”), and RNA binding proteins (“reader”) (Roundtree et al., 2017; Pinello et al., 2018). Numerous studies have indicated that genetic changes in or dysregulated expression of m^6^A regulators contribute to the initiation, malignant progression, and drug resistance of cancers (Wang et al., 2017; Dai et al., 2018; Wang et al., 2018). For instance, METTL3 was reported to promote cell adhension, growth, and progression in PCa through different molecular mechanisms (Cai et al., 2019; Li E. et al., 2020; Yuan et al., 2020). In addition, a recent report indicated that the degradation of IGF2BPs was involved in NSCLC progression (Li et al., 2021). Considering the functional importance of the m^6^A modification in cancers, targeting dysregulated m^6^A methylation regulators may serve as an ideal strategy for cancer therapy in the future.

Although some previous studies have investigated the effect of certain m^6^A regulators on PCa, systematic studies examining m^6^A modification in PCa remain rare. In this study, we aimed to construct a comprehensive diagnostic scoring model, with special focus on epigenomics and transcriptomics. To achieve this, we used The Cancer Genome Atlas (TCGA) PCa cohort to investigate the expression patterns and prognostic value of 17 m^6^A regulators in 551 PCa samples. Additionally, we created a complete atlas of prognosis-related m^6^A regulators, and we found potential regulators that can be used as biomarkers for prognostic stratification. Our study demonstrates the importance of m^6^A regulators in PCa and lays a foundation for the development of new PCa target therapy. Most importantly, we constructed a novel six-gene scoring model which may improve the clinical diagnosis ability of the early stage of PCa patients.

## Materials and Methods

### Data Source and Processing

The raw RNA-seq data and corresponding clinical data of prostate cancer (PCa) were generated within the TCGA (http://cancergenome.nih.gov/). Our study, it should be noted, meets TCGA publication guidelines (Wang et al., 2016). In addition, we also collected other PCa cohorts from ICGC (https://icgc.org/), which included Canada (https://dcc.icgc.org/releases/current/Projects/PRAD-CA) and France (https://dcc.icgc.org/releases/current/Projects/PRAD-FR). The PCa cohorts from China and Britain were out of our consideration due to lack of expression datasets. The overall clinical characteristics of PCa patients are presented in Supplementary Table S1. Here, only 654 PCa patients have both complete follow-up survival information and corresponding expression data, consisting of TCGA-US (N = 492), ICGC-CA (N = 137), and ICGC-FR (N = 25).

**FIGURE 1:**
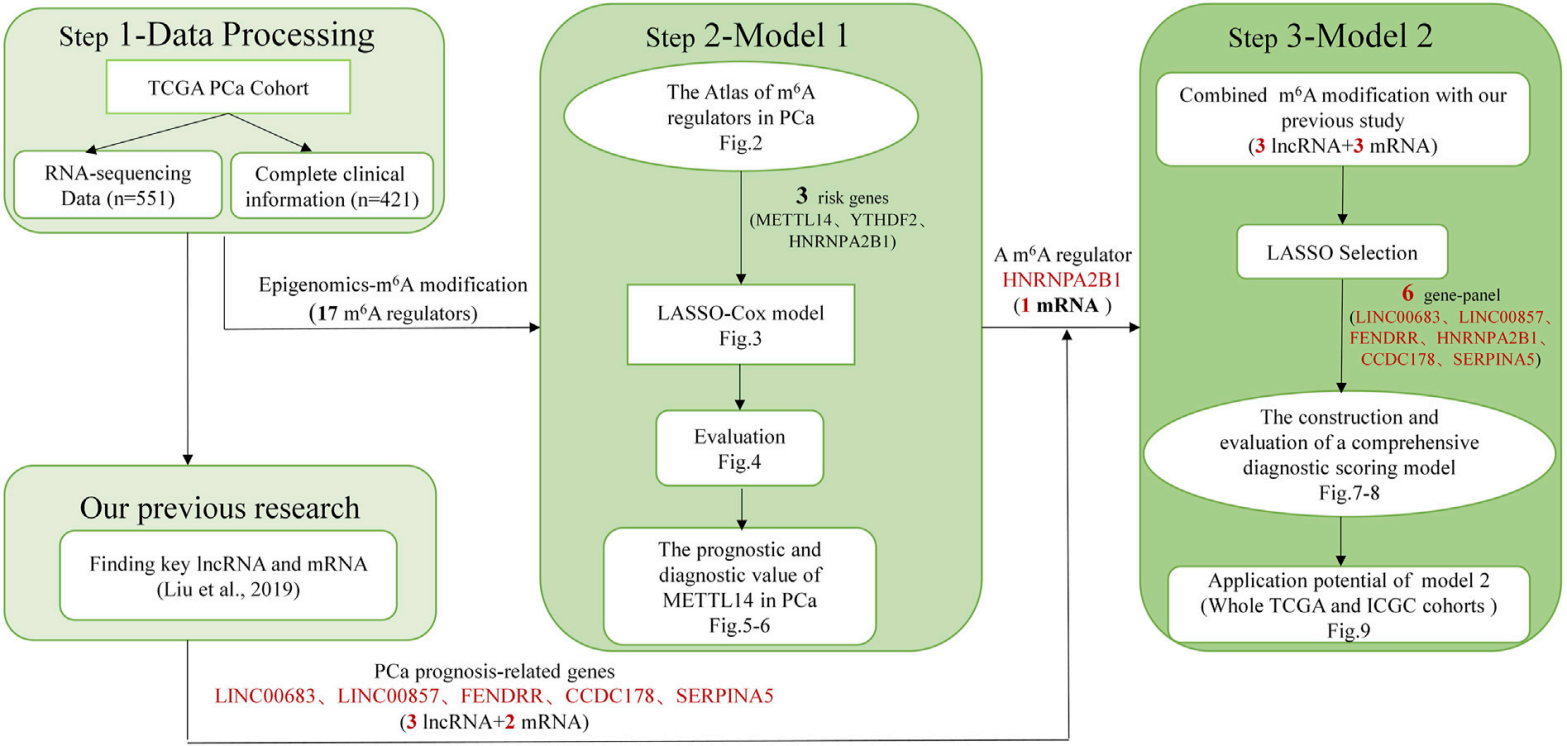
Overall study design.

Details of the overall workflow and the purpose underlying the study design are shown in Figure 1. First, we downloaded 551 samples and clinical information for the PCa cohort that was cross-referenced *via* TCGA categories. Then we systemically analyzed the expression levels and prognostic values of 17 m^6^A regulators in PCa and constructed a LASSO-Cox model using 3 m^6^A regulators. In particular, we also applied the CIBERSORT algorithm to explore the association between m^6^A modification and the tumor microenvironment to further confirm the diagnostic value of prognosis-related m^6^A regulators. Finally, combining with our previous study (Liu et al., 2019), a comprehensive diagnostic scoring model based on a novel 6-gene panel was constructed and further validated by the whole TCGA and ICGC cohorts.

### Identification of Differentially Expressed m^6^A Regulators in PCa

We identified 17 widely reported and verified m^6^A regulators from existing literature and compared the expression levels of these regulators between PCa and normal samples using heat maps and violin plots. Circos Plots generated by using the “RCircos” package indicated the m^6^A regulators in chromosomes (Gu et al., 2014). Spearman analysis was used to show the correlation among these regulators.

### Construction of a LASSO-Cox Diagnostic Scoring Model

To study the prognostic value of these regulators in PCa, univariate analysis was first used to evaluate the correlation between the expression of each risk gene and patient survival. These risk genes were selected to screen and confirm using the least absolute shrinkage and selection operator (LASSO) algorithm (Bovelstad et al., 2007). The best penalty parameter *λ* was determined by using the cross-validation method. The risk score based on these three genes was obtained by using the following formula:

$$\text{Risk}\;\text{Score}=\overset{n}{\underset{i=1}{\sum }}Coef\left(i\right)\times x\left(i\right)$$

Coef(i) represents the coefficient, and x(i) represents the relative expression value of the risk gene through the z-score-transformed. Finally, PCa patients in TCGA were divided into two groups based on the median risk score, and these included the low- and high-risk groups.

### Assessment of the Relevance of Clinical Characteristics

The Kaplan–Meier method with log-rank test was used to evaluate patient survival differences between low-risk groups and high-risk groups. Then, we used a multivariate regression method to determine the impact of each variate on PCa patient survival. Next, ROC curves was applied to validate the accuracy of the model prediction. The area under the curve (AUC) value was calculated by using the R package “survivalROC” (Heagerty et al., 2000). Additionally, we also compared the clinical features (T stage and N) of the two groups (low-risk and high-risk).

### Estimation of Infiltrating Cells Within the Tumor Microenvironment Using the CIBERSORT Algorithm

Numerous studies have suggested that the immune response is significantly associated with the clinical outcome and therapeutic response of cancer patients, particularly in regard to the proportion of immune cells within the microenvironment (Quail and Joyce, 2013; Strasner and Karin, 2015). Based on this, we investigated if a correlation exists between m^6^A regulators and the tumor microenvironment. CIBERSORT is a deconvolution algorithm that can be used to characterize immune cell composition and has been widely used for studying cell heterogeneity (Newman et al., 2015). Therefore, we applied this method to predict the relative proportion of 22 types of infiltrating immune cells in PCa samples. The normalized gene expression data were uploaded to the CIBERSORT website (http://cibersort.stanford.edu/), and the algorithm was run using the LM22 signature and 1,000 permutations. Here, only 172 PCa samples with *p*-value < 0.05 were selected for the analysis.

### Statistical Analysis

Wilcoxon rank sum was performed to test the significance of the infiltration levels of immune cells in PCa samples. Unless otherwise specified in this study, all statistical tests were performed using R 3.5.1 software and GraphPad Prism. The *p* value < 0.05 was considered statistically significant (*p < 0.05, **p < 0.01, and ***p < 0.001).

## Results

### Overview of m^6^A Regulators Profiling in PCa

**FIGURE 2:**
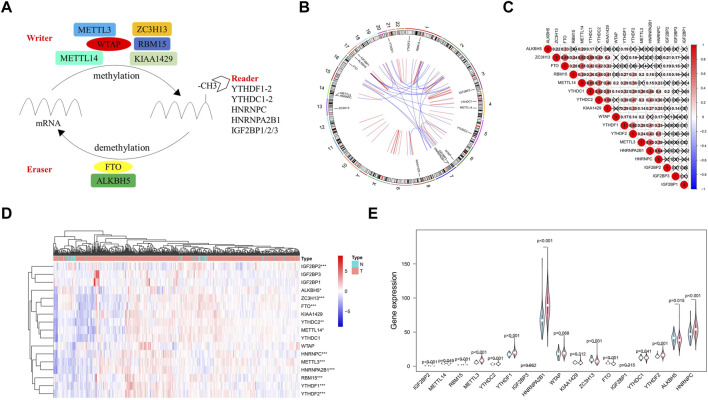
Atlas of m^6^A regulators in prostate cancer (PCa). **(A)** The process of m^6^A modification regulated by different types of m^6^A regualtors. **(B)** The location of m^6^A regulators within chromosomes is illustrated using Circos plots. **(C)** The potential correlation among m^6^A regulators. **(D)** The expression of m^6^A regulators in PCa is shown using a heat map. Red and green represent upregulated genes and downregulated genes, respectively. **(E)** The violin plot of the differentially expression m^6^A regulators between normal samples (light blue) and tumor samples (light red).

According to the current view of m^6^A modifications, this process is dynamically regulated by m^6^A regulators. Here, we initially grouped these methylation regulators into three categories: eraser, writer, and reader (Figure 2A). Then, Circos plots were used to show the details of these m^6^A regulators and their locations on chromosomes. For example, the FTO gene was located on chromosome 16 (Figure 2B). Figure 2C presents the Spearman correlation analysis of 17 m^6^A regulators. With the exception of ALKBH5, ZC3H13, FTO, and IGF2BP families, the relationship between the rest of the m^6^A regulators was positively correlated, and the METTL14 gene and YTHDC1 gene were the most relevant. Considering the important roles of m^6^A modification in cancer initiation, we also compared the expression of 17 m^6^A regulators in 499 PCa samples and 52 normal samples. Compared to levels in normal samples, the expression levels of RBM15, METTL3, YTHDC2, YTHDF1/2, HNRNPC, and HNRNPA2B1 in tumor samples were upregulated, but IGF2BP2 expression levels were downregulated (Figures 2D,E).

### Potential Clinical Utility of Risk Score and m^6^A Regulators

**FIGURE 3:**
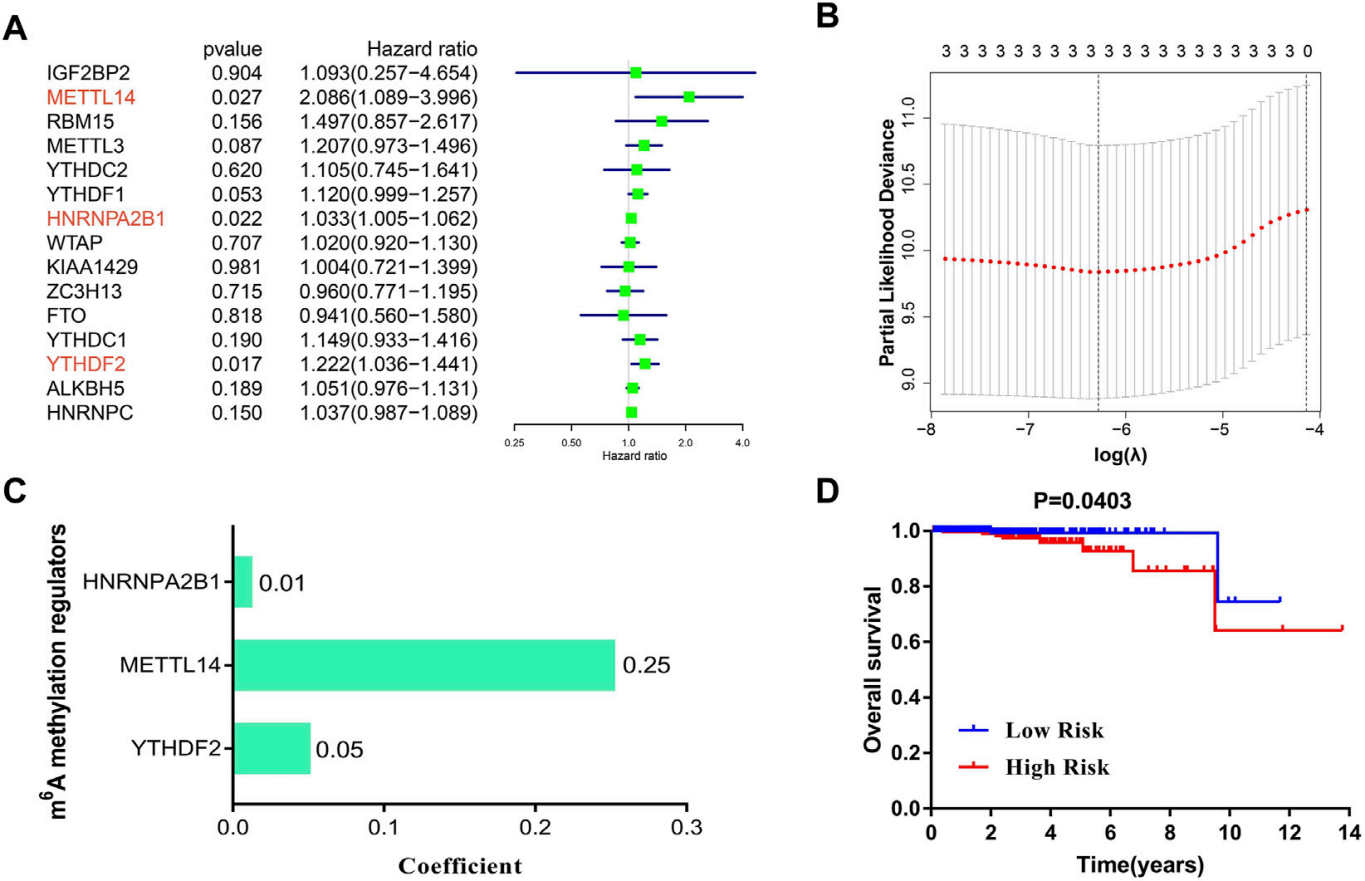
The construction of a LASSO-Cox diagnostic scoring model based on m^6^A regulators. **(A)** Univariate analysis of the 15 regulators on overall survival of PCa patients. **(B,C)** The coefficients of three m^6^A regulators are calculated by using the LASSO algorithm. **(D)** Kaplan–Meier analysis of PCa patients with low-risk (blue) and high-risk (red) groups in TCGA cohorts (N = 492).

In an attempt to establish a diagnostic scoring model, we first performed univariate analysis on the expression levels of 17 m^6^A regulators. The results suggested that METTL14 (HR = 2.09, 95%CI = 1.09–4.00), HNRNPA2B1 (HR = 1.03, 95%CI = 1.01–1.06), and YTHDF2 (HR = 1.22, 95%CI = 1.04–1.44) were significantly correlated with survival of PCa patients (Figure 3A). It should be noted that IGF2BP1(*p-value* = 0.33) and IGB2BP3 (*p-value* = 0.67) were not shown in Figure 3A, because the hazard ratio and confidence interval of these 2 m^6^ regulators were 0. As shown in Figures 3B,C, these three regulators were selected to build a risk score, and coefficients obtained from the LASSO algorithm were used to calculate the risk of each PCa patient. To study the possible prognostic role of the risk score containing three m^6^A regulators, we divided the PCa patients into low- and high-risk groups. The data presented in Figure 3D indicate that the high-risk group exhibited a poorer prognosis.

### Assessment of the Prediction Performance of Risk Score Based on m^6^A Regulators

**FIGURE 4:**
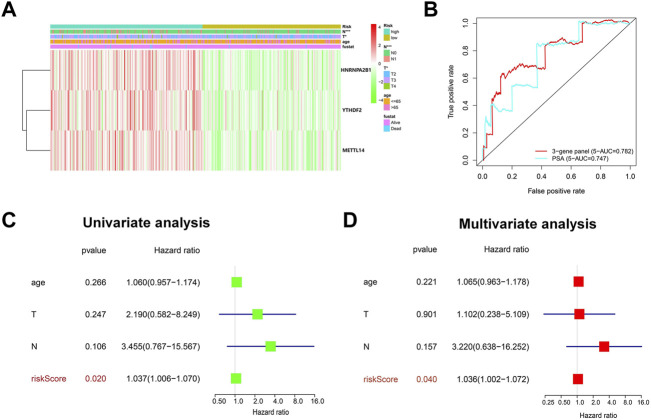
Evaluation of the prediction performance of the m^6^A prognostic scoring model. **(A)** Heatmap showing the differences in the clinicopathologic features of PCa patients with low-risk group and high-risk groups. **(B)** The predictive efficiency of the 3-gene panel and PSA were shown by a 5-year ROC curve. **(C,D)** Univariate and multivariate analysis of the correlation between clinicopathological features and overall survival of PCa patients.

We next investigated the correlation between the three m^6^A regulators and clinicopathological features of PCa, including age, T-status, and N-status. The heatmap displays the expression level of these m^6^A regulators in the low-risk and high-risk groups (Figure 4A). We found that compared to the low-risk group, the high-risk group was closely related to higher T-status (T3/T4) and N-status (N1) (Figure 4A). ROC was then used to predict 5-year survival for PCa patients. The data presented in Figure 4B indicate that 3-gene panel risk scores (AUC = 0.782) exhibit relatively higher prediction accuracy than PSA (AUC = 0.747). Finally, univariate and multivariate analyses were used to evaluate the possibility of using the risk score as an independent prognostic factor. Both results revealed that the risk score was correlated with overall survival (Figures 4C,D). Based on these data, there is a strong correlation between risk scores and clinicopathological features of PCa.

### The Prognostic Value of METTL14 for PCa Patients

**FIGURE 5:**
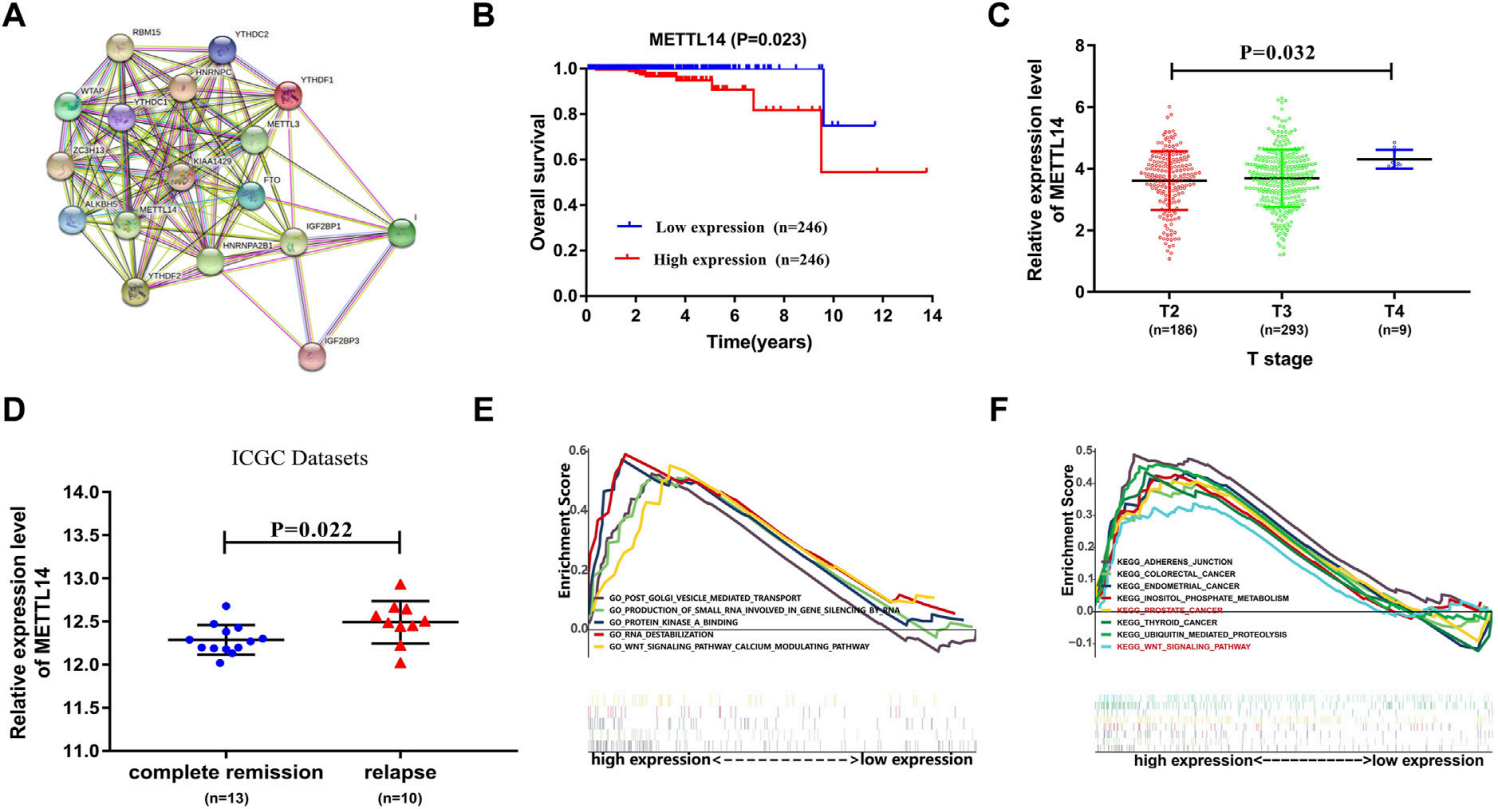
Underlying prognostic value of METTL14 in PCa. **(A)** Protein–protein interaction network of 17 m^6^A regulators in which the key role of METTL14 is shown. **(B)** Kaplan–Meier analysis of METTL14 with low expression (blue) and high expression (red) in PCa patients. **(C,D)** The association of METTL14 expression with different tumor stages and disease status. **(E,F)**. Gene set enrichment analysis for TCGA PCa samples with high expression of the METTL14 signature.

Furthermore, we constructed a protein–protein interaction network of 17 m^6^A regulators using Cytoscape software (Figure 5A) (Su et al., 2014). The degree of METTL14 was 14 among the three prognosis-related m^6^A regulators (Supplementary Table S2). To further study the possible role of METTL14 in PCa, we performed an overall survival analysis and a clinical features correlation analysis. The expression of METTL14 was significantly related to the overall survival of PCa patients and to tumor stage T. Additionally, we found that the expression level of METTL14 is associated with disease status (Complete regression/relapse) according to ICGC datasets. As shown in Figures 5B–D, patients in the high-expression group exhibited reduced overall survival and higher T-status and relapse rate.

The TCGA PCa cohort was also divided into high-expression and low-expression groups based on median METTL14 expression. Gene set enrichment analysis (GSEA) was then used to find potential associated signaling pathways and biological processes. Interestingly, we found that a number of cancer-related pathways such as Wnt pathways, KEGG Prostate Cancer, KEGG Colorectal Cancer, and KEGG Thyroid Cancer were predominantly enriched in the high-expression group. This implied that elevated expression of METTL14 may contribute to unfavorable prognosis in PCa (Figures 5E,F).

### METTL14 May Be a Potential Indicator for Tumor Microenvironment Modulation

**FIGURE 6:**
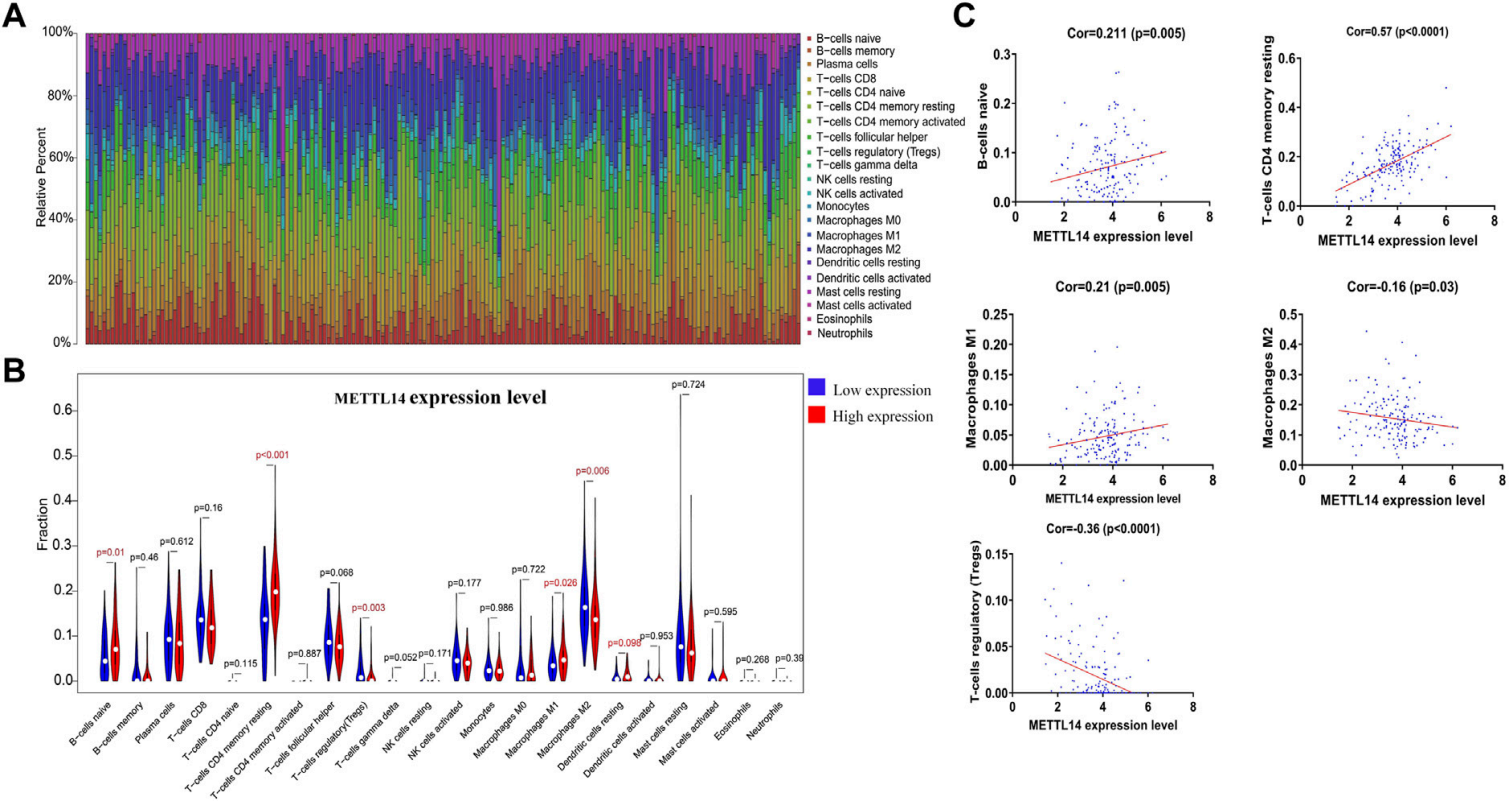
Potential association between METTL14 expression and the tumor microenvironment. **(A)** Bar plots showing the proportion of 22 specific immune cells in each of the PCa samples. **(B)** Violin plot depicting the difference of immune infiltration in PCa samples with low or high METTL14 expression. **(C)** Pearson correlation analysis of five types of immune cell fractions with METTL14 expression (*p* < 0.05).

To further verify the diagnostic value of prognosis-related m^6^A regulators, we investigated the potential correlation between METTL14 expression and the tumor microenvironment. Using the CIBERSORT algorithm (Newman et al., 2015), we created a landscape of the PCa tumor microenvironment. Within this landscape, 22 specific immune cell fractions in each PCa sample are shown in a boxplot (Figure 6A). Interestingly, the infiltration levels of naive B-cells, CD4^+^ memory resting T-cells, M1 macrophages, and dendritic resting cells in the METTL14 high-expression group were higher than those in the low-expression group (Figure 6B). In contrast, the infiltration levels of M0 macrophages and regulatory T-cells were lower in the METTL14 high-expression group (Figure 6B). Additionally, we found that three types of immune cells were positively correlated with METTL14 expression, and these included naive B-cells, CD4^+^ memory resting T cells, and M1 macrophages. Two types of immune cells were negatively correlated with METTL14 expression, and these included regulatory T-cells and M2 macrophages. These data further confirmed that the expression levels of METTL14 affected the immune activity of the tumor microenvironment in PCa (Figure 6C).

### The Construction and Evaluation of a Comprehensive Diagnostic Scoring Model

As previously mentioned, although we constructed a m^6^A prognostic scoring model, its prediction performance may not be good enough for PCa diagnosis. Therefore, we further constructed a comprehensive diagnostic scoring model to improve the predictive ability. Here, more biomarkers related to PCa prognosis were taken into consideration. Combined with our previous research results, in which we found three lncRNAs (LINC00683, LINC00857, and FENDRR) and two mRNAs (CCDC178 and SERPINA5) were significantly related with overall survival of PCa patients (Liu et al., 2019), and 3 m^6^A regulators (METTL14, HNRNPA2B1, and YTHDF2) were combined with five RNAs (2lncRNA+3mRNA) in a variety of ways in order to construct an optimal complex model. Finally, HNRNPA2B1 plus five RNAs was the best model combination we found from seven different ways (Supplementary Table S3).

**FIGURE 7:**
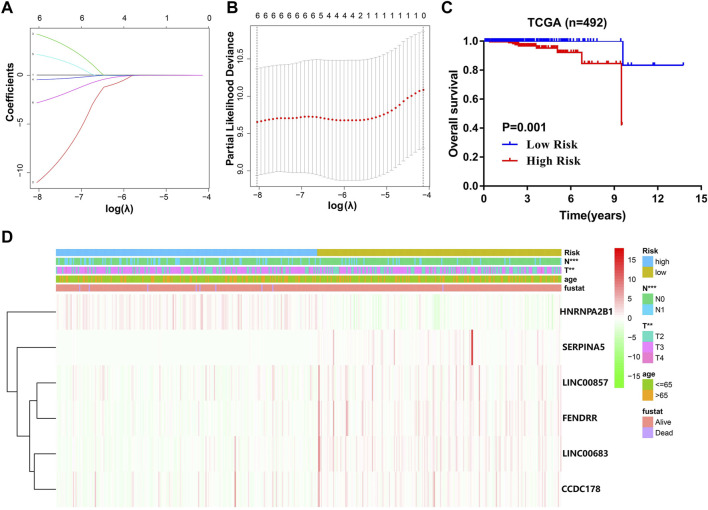
Construction of a comprehensive diagnostic scoring model for PCa. **(A,B)** The process of selecting risk genes using the LASSO algorithm. **(C)** Kaplan–Meier analysis of PCa patients for high-risk (red) and low-risk groups (blue). **(D)** Heatmap of differences in the clinicopathologic features of PCa patients for low-risk and high-risk groups.

**FIGURE 8:**
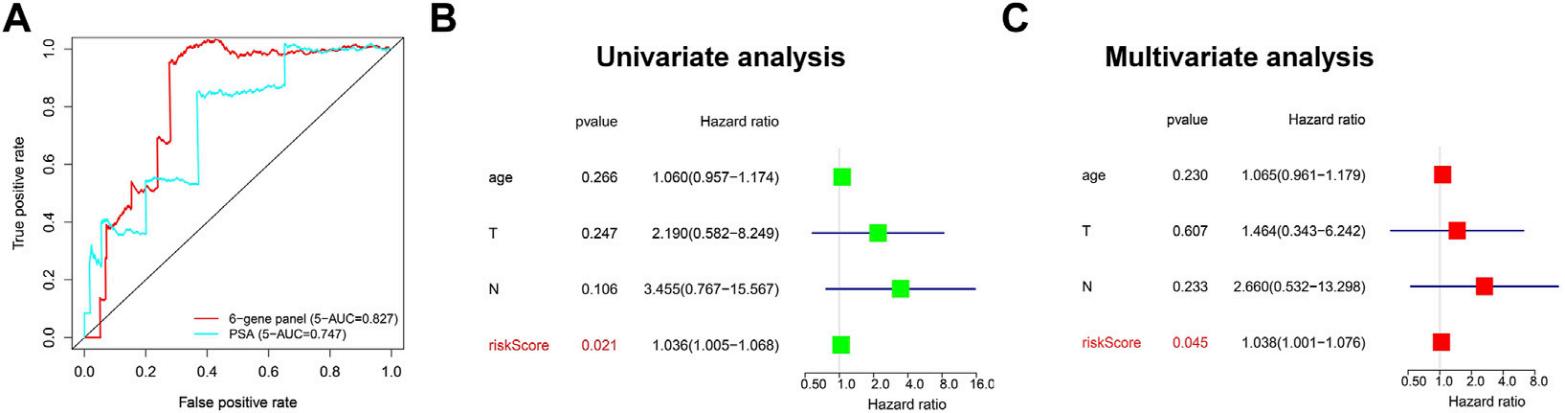
Evaluation of the prediction performance of the 6-gene panel diagnostic scoring model in TCGA cohorts. **(A)** The predictive efficiency of the 6-gene panel model and PSA were shown by a 5-year ROC curve. **(B,C)** Univariate and multivariate analysis of the correlation between clinicopathological features and overall survival of PCa patients.

The data presented in Figure 7 indicated that the scoring model based on the six-gene panel was effective for the prediction of PCa prognosis. By LASSO algorithm selection, six genes (HNRNPA2B1, LINC00683, LINC00857, FENDRR, CCDC178, and SERPINA5) were retained (Figures 7A,B). Based on this model, we were surprised to find that the overall survival of PCa patients in the high-risk group exhibited significantly reduced and closely related to high T-status (T3/T4) and N-status (N1) (Figures 7C,D; Table 1). Besides, the accuracy of model prediction and the clinical diagnosis feasibility were validated by the 5-year ROC curve and independent prognosis analysis (Figure 8). Compared with the PSA, 3 m^6^A regulators scoring model, and previous established model (Leyten et al., 2015; Shao et al., 2020; Wang et al., 2020), the 6-gene panel scoring model has the best predictive performance (AUC = 0.827) (Figure 8; Table 1). These results indicated that the 6-gene panel risk score has the potential to be used as an independent prognostic factor in the treatment and diagnosis of PCa patients.

**TABLE 1:** Comparison of prediction performance between our scoring model and the previous established model.

Prediction ability	Gene signature	Significance of difference of overall survival^a^	Significance of difference of clinical T status^b^	AUC value of ROC curve
Model				
Leyten et al	HOXC6, TDRD1, DLX1, sPCA3^c^	—	—	0.81
Shao et al.^d^	ZNF467, SH3RF2, PPFIA2, MYT1, TROAP, GOLGA7B	*p* < 0.001 (TCGA)	—	0.73 (TCGA)
		*p* = 0.003 (GEO)		0.76 (GEO)
		*p* < 0.001 (FUSCC)		0.72 (FUSCC)
		*p* < 0.001 (TAHNU)		0.81 (TAHNU)
Wang et al.	METTL14, YTHDF2	*p* = 0.001	—	0.762
m6A prognostic scoring model	METTL14, YTHDF2, HNRNPA2B1	*p* = 0.040	T (*)	0.782
6-gene panel scoring model	LINC00683, LINC00857, FENDRR, HNRNPA2B1, CCDC178, SERPINA5	*p* = 0.001 (Training set)	T (**)	0.827 (Training set)
		*p* = 0.0005 (Testing set)	(Training set)	0.898 (Testing set)

a Significance of difference of Overall Survival between low-risk groups and high-risk groups.

b Clinical characteristics significant differences between low-risk groups and high-risk groups (*p < 0.05, **p < 0.01).

c sPCA3 is serum prostate-specific antigen.

d The Cancer Genome Atlas (TCGA), Gene Expression Omnibus (GEO) serve as discovery set and test set separately, the databases of Fudan University Shanghai Cancer Center (FUSCC) and Third Affiliated Hospital of Nantong University (TAHNU) were an external validation set.

### Validation of Six-Gene Panel Scoring Model in Large-Scale PCa Cohorts

**FIGURE 9:**
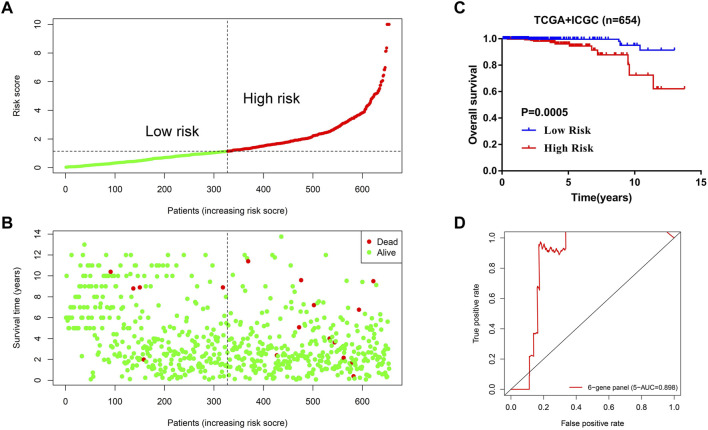
Application potential of the 6-gene panel scoring model in large-scale PCa cohort diagnosis. **(A,B)** The distribution of risk score and survival status of PCa patients in the whole TCGA and ICGC cohorts (N = 654). **(C)** Kaplan–Meier analysis of PCa patients for high-risk (red) and low-risk groups (blue). **(D)** The predictive efficiency of the 6-gene panel scoring model was assessed by using a 5-year ROC curve.

To test the robustness and the application potential of the six-gene panel scoring model in PCa diagnosis, we integrated whole TCGA and ICGC cohorts containing three different countries: America (TCGA-US), Canada (ICGC-CA), and France (ICGC-FR). We first constructed a multivariate Cox regression model using these six genes (Supplementary Figure S1) and then calculated the risk score of 654 PCa patients. The risk score distribution and survival status of PCa patients were shown in Figures 9A,B. For the whole TCGA and ICGC cohorts (N = 654), we observed that the overall survival of PCa patients in the high-risk group were also significantly reduced (Figure 9C; Table 1), which is consistent with the trends using the training dataset (TCGA-US). In addition, the model prediction ability was verified again by the 5-year ROC curve (AUC = 0.898). It still showed good performance for predicting prognosis in large-scale PCa cohorts (Figure 9D; Table 1).

## Discussion

PCa is asymptomatic in the early stages of disease development, thus leading to difficulties in diagnosing this disease. However, advanced PCa that progresses to tumor metastases is usually regarded as incurable or difficult to treat (Ardura et al., 2020). Current clincial diagnosis largely depends on PSA screening; however, this often fails to detect certain aggressive tumors and can lead to overtreatment in a considerable number of patients (Lilja et al., 2008; Scott and Munkley, 2019). Therefore, there is an urgent need to identify novel biomarkers that can be used for clinical diagnosis. As PCa is a heterogeneous and multifocal disease, multiple biomarkers are required to aid in directing clinical decisions (Scott and Munkley, 2019). In order to combine liquid biopsy for effective diagnosis, we aimed to explore new biomarkers for PCa from the epigenomics and transcriptomics perspective, and we hope to construct a comprehensive scoring model that can be used in clinical diagnostics.

Over the past few years, m^6^A modification has been demonstrated to play a vital role in major bioprocesses such as self-renewal, tissue development, control of circadian rhythms, primary mRNA processing, and RNA–protein interactions (Wang et al., 2014; Liu et al., 2015; Meyer et al., 2015; Zhao et al., 2017). More recently, a number of efforts have been devoted to investigate the biological impacts and the associated machinery of dysregulated m^6^A modification in the context of various cancers, including PCa (Li et al., 2017; Wang et al., 2017; Wu et al., 2018). These studies suggested that up- or down-regulation of particular m^6^A regulators is associated with tumors, and the same m^6^A regulators may exert different functions in different cancers.

In the present study, we attempted to delineate a complete atlas of prognosis-related m^6^A regulators for PCa and develop a new prognostic prediction scoring model. Here, only 17 widely reported and verified m^6^A regulators were taken into consideration. First, the atlas of m^6^A regulators in PCa was mapped based on TCGA RNA-sequencing data, including categories, chromosome location, correlation, and expression level (Figure 2). Finally, three PCa prognosis-related m^6^A regulators (METTL14, HNRNPA2B1, and YTHDF2) were identified by univariate analysis, and a LASSO-Cox model was constructed based on these regulators (Figure 3). Both multivariate Cox analysis and ROC analysis revealed that the 3-gene panel risk scores can predict clinicopathlogical features of PCa as an independent prognostic factor (Figure 4). In order to improve the prediction performance of clinical diagnosis, we next constructed a comprehensive diagnostic scoring model. Here, we combined five RNAs reported in our previous study (Liu et al., 2019) and 3 m^6^A regulators found in this study in seven different ways (Supplementary Table S3). Finally, we found HNRNPA2B1 plus five RNAs was the best model combination. This six gene-panel risk signature is significantly correlated with advanced clinical features of malignancy (T3/T4, N1), indicating that we can distinguish between early and advanced PCa patients according to this model (Figure 7).

To better illustrate our results, we conducted literature curation for three risk genes that construct the m^6^A prognostic scoring model. Among these prognosis-related m^6^A regulators, a number of previous studies have indicated that YTHDF2 is primarily involved in the malignant progression of pancreatic cancer, hepatocellular carcinoma, and acute myeloid leukemia (Chen et al., 2017; Paris et al., 2019; Zhong et al., 2019). The findings by Li et al. revealed that YTHDF2 was involved in the development of PCa by targeting miR-493–3p or inducing AKT phosphorylation (Li et al., 2017; Li et al., 2020). HNRNPA2B1 was previously reported to be associated with a number of cancers, including liver cancer, breast cancer, lung cancer, cervical cancer, and pancreatic cancer (Barcelo et al., 2014; Chen et al., 2017; Hu et al., 2017; Hung et al., 2017; Shi et al., 2018). Although Li et al. revealed that HNRNPA2B1 was associated with the overall survival of PCa patients, the function and role of HNRNPA2B1 in the context of PCa were not clear (Li et al., 2019). In regard to METTL14, currently available studies have demonstrated that it was significantly correlated with hepatocellular carcinoma, glioblastoma, acute myeloid leukemia, gastric cancer, and breast cancer (Cui et al., 2017; Ma et al., 2017; Weng et al., 2018; Wu et al., 2019; Zhang et al., 2019). So far, only two studies explored the possible roles of METTL14 in the context of PCa (Panneerdoss et al., 2018; Wu et al., 2021). One found that the depletion of METTL14 could inhibit clonability and migration of PCa (DU-145) cells, while another showed METTL14 was negatively correlated with the Gleason grade in PCa. Despite this, the underlying mechanism of METTL14 in PCa remains unclear. In our analysis results, METTL14 was proved to possess important clinical prognostic value based on large-scale PCa cohorts (Figure 5). Interestingly, METTL14 expression affected the immune activity of the tumor microenvironment, thus providing additional insight into the therapeutics of PCa (Figure 6). Combining our existing data with the previous research, we believed that METTL14 may prove to be a promising biomarker and therapeutic target in PCa.

Since the incidence and mortality of PCa have increased in recent years, it will be of great importance to develop new therapeutic approaches for this disease based on promising prognostic and diagnostic biomarkers. In this study, we finally constructed a comprehensive diagnostic scoring model based on a novel 6-gene panel. The model showed strong robust performance with respect to predicting PCa prognosis (Figure 8). Compared with the results of other research group (Leyten et al., 2015; Shao et al., 2020; Wang et al., 2020), the prediction performance of the novel 6-gene panel prognostic model (AUC = 0.827, Table 1) is relatively higher than their models. Most importantly, the application potential of the novel 6-gene panel prognostic model was further verified in large-scale PCa cohorts (Figure 9). In addition, using the RMVar database, we also found that 5 (LINC00683, LINC00857, FENDRR, SERPINA5, and CCDC178) out of the six genes could be methylated by m^6^A modification, but the direct evidence showing that “reader” HNRNPA2B1 could regulate the methylation level of these five genes is lacking within medium- or high-confidence experiment levels (Luo et al., 2021). It should be pointed out that HNRNPA2B1 itself could also be methylated by m^6^A modification based on the m^6^A-Atlas database (Tang et al., 2021). Therefore, the potential regulatory relationship between these six genes and m^6^A modification may need to be explored further by experiments in the future.

In summary, we expect that the application of this novel 6-gene panel scoring model will not only contribute to the selection of an appropriate therapeutic strategy, enabling precise prediction of personal prognosis, but will also further promote the understanding of the basic biology of PCa. Although further biological experiments are required to validate our findings, we believe that this model could also be used in the future as a complement to PSA screening for PCa (Figure 8).

## Data Availability

The datasets analyzed in this study are available from the TCGA database (http://cancergenome.nih.gov/) and the ICGC database (https://icgc.org/).
