# Supplementary material for: Construction of a Comprehensive Diagnostic Scoring Model for Prostate Cancer Based on a Novel Six-Gene Panel
Source: Front Genet. 2022 Apr 26;13:831162. doi: 10.3389/fgene.2022.831162 (PMC9086319; doi:10.3389/fgene.2022.831162)
Supplement: Supplementary file 1 [file DataSheet1.docx]

**Table S1:Detailed clinical information of PCa patients in this study**

|  | | **PCa Datasets** | | |  |
| --- | --- | --- | --- | --- | --- |
| **Variables** | | **TCGA-US** | **ICGC-CA** | **ICGC-FR** |  |
|  | | **(N=421)** | **(N=375)** | **(N=25)** |  |
| **Age** | ≤65 | 125 | 185 | 16 |  |
|  | >65 | 296 | 190 | 9 |  |
| **T stage** | T1 | **/** | 178 | 11 |  |
|  | T2 | 144 | 196 | 14 |  |
|  | T3 | 267 | 1 | **/** |  |
|  | T4 | 10 | **/** | **/** |  |
| **N stage** | N0 | 342 | **/** | 25 |  |
|  | N1 | 79 | **/** | **/** |  |
| **Status** | Complete remission | **/** | **/** | 13 |  |
|  | Relapse | **/** | **/** | 10 |  |
|  |  |  |  |  |  |
| **RNA-sequencing data** | | 499 | 144 | 25 |  |

**Table S2: The degree of 17 m^6^A regulators**

| Gene | Degree |
| --- | --- |
| HNRNPA2B1  KIAA1429  METTL3  ALKBH5  FTO  HNRNPC  IGF2BP1  METTL14 | 16  15  15  14  14  14  14  14 |
| YTHDC1  YTHDC2  YTHDF1  YTHDF2 | 14  14  14  14 |
| ZC3H13  RBM15  WTAP | 14  14  14 |
| IGF2BP2  IGF2BP3 | 8  3 |

**Table S3: Comparison of prediction performance of 7 different model combination ways.**

|  | The retained gene after LASSO selection | Significance of difference of Overall Survival | AUC value of ROC curve |
| --- | --- | --- | --- |
| 5 RNAs + 3 m^6^A regulators (METTL14, YTHDF2 andHNRNPA2B1) | METTL14, YTHDF2 and HNRNPA2B1 | P=0.0403 | 0.782 |
| 5 RNAs + 2 m^6^A regulators (METTL14 and YTHDF2) | METTL14 and YTHDF2 | P=0.145 | 0.762 |
| 5 RNAs + 2 m^6^A regulators (METTL14 and HNRNPA2B1) | METTL14 and HNRNPA2B1 | P=0.0271 | 0.783 |
| 5 RNAs + 2 m^6^A regulators (YTHDF2 and HNRNPA2B1) | YTHDF2 and HNRNPA2B1 | P=0.0481 | 0.732 |
| 5 RNAs + 1 m^6^A regulators (METTL14) | HNRNPA2B1, LINC00683, LINC00857, FENDRR, CCDC178 and SERPINA5 | P=0.00231 | 0.798 |
| 5 RNAs + 1 m^6^A regulators (YTHDF2) | YTHDF2 | P=0.457 | 0.71 |
| 5 RNAs + 1 m^6^A regulators (HNRNPA2B1) | HNRNPA2B1, LINC00683, LINC00857, FENDRR, CCDC178 and SERPINA5 | P=0.00103 | 0.827 |

Table S4 Comparison of prediction performance between our scoring model and previous established model

| Prediction  ability Model | Gene signature | Significance of difference of Overall Survival***^1^*** | Significance of difference of clinical T Status***^2^*** | AUC value of ROC curve |
| --- | --- | --- | --- | --- |
| *Leyten et al.* | HOXC6, TDRD1, DLX1, sPCA3***^3^*** | */* | / | 0.81 |
| Shao *et al****^4^****.* | ZNF467, SH3RF2, PPFIA2, MYT1, TROAP, GOLGA7B | *P*<0.001(TCGA) *P*=0.003 (GEO) *P*<0.001 (FUSCC) *P*<0.001 (TAHNU) | / | 0.73 (TCGA)  0.76 (GEO)  0.72 (FUSCC)  0.81 (TAHNU) |
| Wang *et al.* | METTL14, YTHDF2. | *P*=0.001 | / | 0.762 |
| m^6^A prognostic scoring model | METTL14, YTHDF2, HNRNPA2B1. | *P*=0.040 | T (*) | 0.782 |
| 6-gene panel scoring model | LINC00683, LINC00857, FENDRR, HNRNPA2B1, CCDC178, SERPINA5. | *P*=0.001(Training set)  *P*=0.0005 (Testing set) | T (**) (Training set) | 0.827(Training set) 0.898 (Testing set) |

1. Significance of difference of Overall Survival between low-risk groups and high-risk groups
2. Clinical characteristics significant differences between low-risk groups and high-risk groups (* P < 0.05, ** P < 0.01)
3. sPCA3 is serum prostate-specific antigen.
4. The Cancer Genome Atlas (TCGA), Gene Expression Omnibus (GEO) serve as discovery set and test set separately, the databases of Fudan University Shanghai Cancer Center (FUSCC) and Third Affiliated Hospital of Nantong University (TAHNU) were an external validation set.


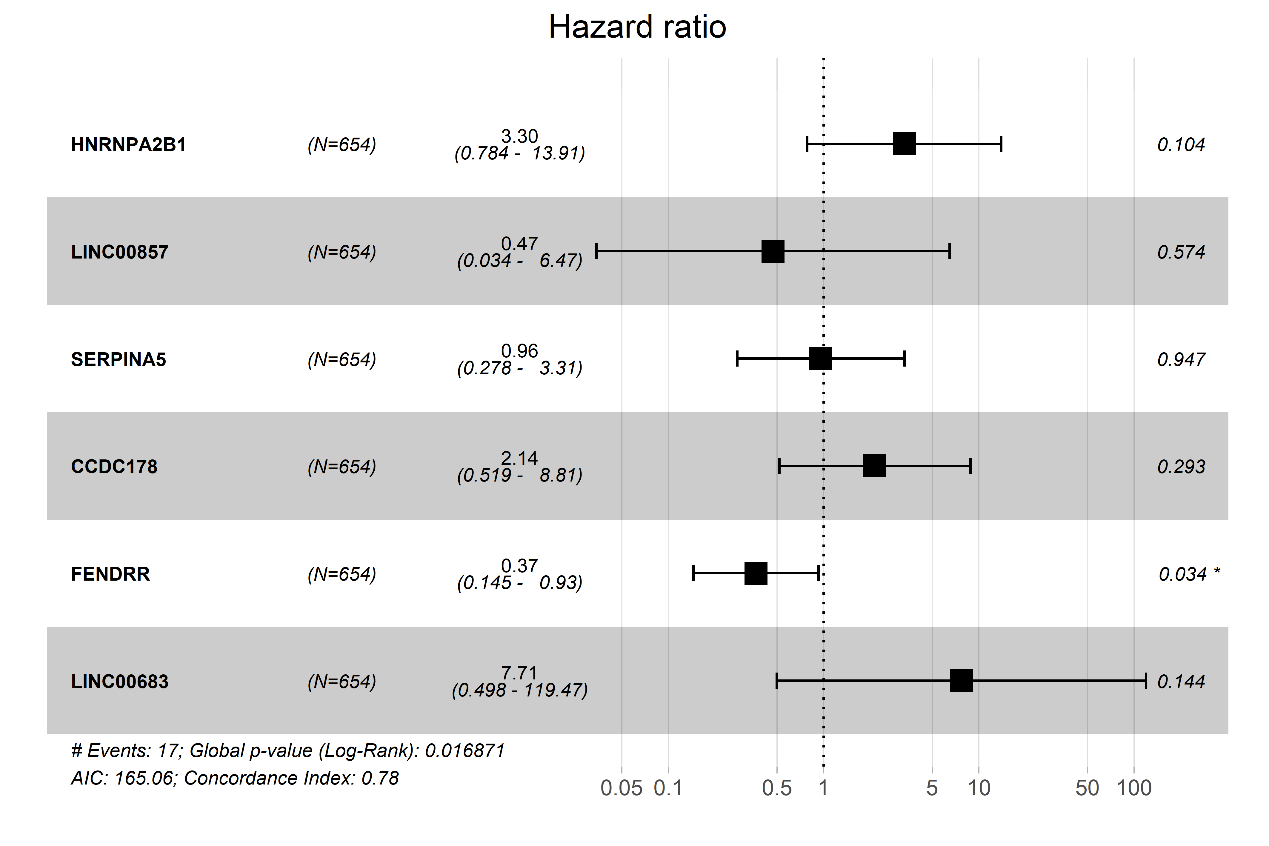


**Figure S1: Multivariate cox regression model for six gene in large-scale PRAD cohorts**
